# Prediction of Breast Cancer Response to Neoadjuvant Therapy with Machine Learning: A Clinical, MRI-Qualitative, and Radiomics Approach

**DOI:** 10.3390/life15081165

**Published:** 2025-07-23

**Authors:** Rami Hajri, Charles Aboudaram, Nathalie Lassau, Tarek Assi, Leony Antoun, Joana Mourato Ribeiro, Magali Lacroix-Triki, Samy Ammari, Corinne Balleyguier

**Affiliations:** 1 Imaging Department, Gustave Roussy Cancer Campus, Université Paris-Saclay, 94805 Villejuif, France; 2 Biomaps, UMR1281, Université Paris-Saclay, 94805 Villejuif, France; 3 Department of Medical Oncology, Gustave Roussy, Université Paris-Saclay, 94805 Villejuif, France; 4 Department of Pathology, Gustave Roussy, Université Paris-Saclay, 94805 Villejuif, France

**Keywords:** radiomics, machine learning, breast cancer, pathological complete response, neoadjuvant chemotherapy

## Abstract

Background: Pathological complete response (pCR) serves as a prognostic surrogate endpoint for long-term clinical outcomes in breast cancer patients receiving neoadjuvant systemic therapy (NAST). This study aims to develop and evaluate machine learning-based biomarkers for predicting pCR and recurrence-free survival (RFS). Methods: This retrospective monocentric study included 235 women (mean age 46 ± 11 years) with non-metastatic breast cancer treated with NAST. We developed various machine learning models using clinical features (age, genetic mutations, TNM stage, hormonal receptor expression, HER2 status, and histological grade), along with morphological features (size, T2 signal, and surrounding edema) and radiomics data extracted from pre-treatment MRI. Patients were divided into training and test groups with different MRI models. A customized machine learning pipeline was implemented to handle these diverse data types, consisting of feature selection and classification components. Results: The models demonstrated superior prediction ability using radiomics features, with the best model achieving an AUC of 0.72. Subgroup analysis revealed optimal performance in triple-negative breast cancer (AUC of 0.80) and HER2-positive subgroups (AUC of 0.65). Conclusion: Machine learning models incorporating clinical, qualitative, and radiomics data from pre-treatment MRI can effectively predict pCR in breast cancer patients receiving NAST, particularly among triple-negative and HER2-positive breast cancer subgroups.

## 1. Introduction

Breast cancer remains the most commonly diagnosed malignant tumor worldwide, with over 2 million new cases diagnosed annually [1]. Recent years have witnessed significant changes in patient management, leading to improved prognosis, with neoadjuvant systemic therapy (NAST) playing a crucial role [2]. NAST, defined as the administration of systemic therapy before definitive breast surgery, effectively downstages disease and increases breast-conserving surgery opportunities [3]. Additionally, it enables in vivo assessment of treatment sensitivity and provides valuable prognostic information [4].

The treatment paradigm for early-stage breast cancer has progressively shifted from adjuvant to neoadjuvant settings, with expanding NAST indications. Pathological complete response (pCR), assessed on surgical specimens following neoadjuvant treatment, has emerged as a surrogate endpoint correlating with long-term clinical outcomes [5]. Evidence demonstrates that breast cancer patients achieving pCR following active therapy experience improved survival, particularly in aggressive tumor subtypes [5]. Furthermore, pCR serves as a validated biomarker for determining post-operative therapeutic sequences, particularly in triple-negative and HER2-positive breast cancer subtypes [6,7].

Optimizing pCR prediction would enable better patient stratification for treatment tailoring [8], identify non-responders to minimize toxicity, and potentially avoid surgery in specific cases [9]. Various methods have been employed to predict pCR, including clinical examination and radiological analysis through ultrasound, mammography, or MRI [10,11]. Post-treatment MRI-based methods are most commonly evaluated but sometimes remain inconclusive [12]. Dynamic contrast-enhanced (DCE) MRI represents the most accurate method for evaluating NAST response, with studies investigating various parameters such as ultrafast DCE [13] and associations between tumor response and background parenchymal enhancement [14].

Radiomics represents a rapidly expanding research field in medical imaging with encouraging results [15,16]. This approach involves extracting multiple quantitative descriptors from tumor segmentation in medical images. Radiomics features of a tumor are hypothesized to link to its phenotype and, consequently, to gene expression patterns [17]. Furthermore, 3D imaging analyzes the entire tumor volume, whereas biopsy is susceptible to sampling errors due to tumor heterogeneity [18]. Therefore, in the era of personalized medicine, radiomics could serve as a valuable complementary tool to genomics and molecular analysis [19].

Machine learning (ML) enables the processing of the extensive data generated through radiomics [20]. Several studies combining radiomics from pre-treatment breast MRI and machine learning have been reported [21,22,23,24,25]. However, these studies often present limitations that complicate clinical integration and reproducibility, including manual segmentation [22], limited MRI models [23], feature extraction with proprietary software [24], and insufficient data on clinical outcome prediction [25].

In this study, we conducted a “real-world” radiomics evaluation in breast cancer patients [26], with most patients referred to our institution having previously undergone MRI. The 235 included patients were divided into training and test groups with different MRI models to demonstrate the algorithms’ resilience to machine variations. We employed commercial software for semi-automatic segmentation and feature extraction to approximate clinical workflow conditions. Furthermore, we performed additional analyses, previously unreported in the literature, comparing radiomics ML models with MRI qualitative and clinical ML models (preferring simpler models when feasible) and evaluating our models against the current standard technique for pCR prediction–post-treatment MRI response. Our aim was to develop algorithms based on clinical records, MRI-derived qualitative and quantitative data to predict both pCR and recurrence-free survival (RFS), evaluate which data most significantly impacted ML model performance, and compare their performance to post-neoadjuvant therapy MRI features.

## 2. Materials and Methods

### 2.1. Cohort Description

We retrospectively enrolled a total of 307 consecutive female patients, aged 18 years or older, diagnosed with histologically confirmed, non-metastatic invasive breast cancer and treated with neoadjuvant systemic therapy followed by surgery at our tertiary cancer center between April 2014 and August 2018. All included patients had undergone baseline clinical assessment, biopsy with immunohistochemical profiling (ER, PR, HER2, Ki67), and pre-treatment breast MRI prior to initiating NAST. Neoadjuvant therapy protocols followed contemporary national and institutional guidelines, including chemotherapy with or without targeted agents, depending on molecular subtype.

**Figure 1:**
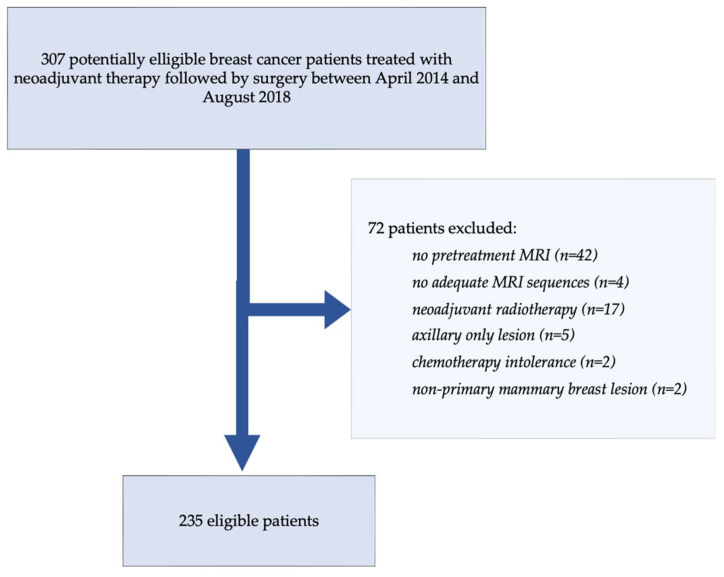
Visual cohort selection process with exclusion criteria.

A total of 72 patients were excluded from the study cohort based on the following predefined criteria: (a) unavailable pre-treatment breast MRI data (*n* = 42), (b) inadequate MRI image quality of incomplete required sequences (*n* = 4), (c) administration of neoadjuvant radiotherapy (*n* = 17), (d) cases involving isolated axillary disease without detectable primary breast tumor (*n* = 5), (e) premature discontinuation of chemotherapy due to intolerance or adverse events (*n* = 2), and (f) tumors determined to be of non-primary mammary lesions (*n* = 2). The final cohort included 235 patients with complete and usable pre-treatment and follow-up data, as shown in Figure 1. Baseline clinical, pathological, and demographic characteristics are summarized in Table 1.

All patients received written notification of their study enrollment. In accordance with national regulations on data privacy and research ethics, the study protocol was reviewed and approved by the institutional review board and complied with the General Data Protection Regulation (GDPR). This study was also declared to the National Commission of Freedom and Informatics and Health Data Hub.

### 2.2. Data Sources and Feature Extraction

To develop and evaluate our predictive models, we incorporated three distinct but complementary data sources for each patient: (1) clinical data derived from medical records, (2) morphological imaging features extracted from expert radiological interpretation of pre-treatment breast MRIs, and (3) high-dimensional radiomic features extracted from tumor segmentation. This multimodal data integration aimed to reflect real-world clinical workflows and maximize the biological and imaging information available for model training.

#### 2.2.1. Clinical Features

Clinical variables were retrieved retrospectively from electronic medical records and pathology reports by two researchers under the supervision of a medical oncologist and a radiologist. The following variables were collected for each patient at the time of diagnosis:

- Age at diagnosis (in years, as a continuous variable);
- Menopausal status (pre- or postmenopausal);
- Tumor histological type (e.g., invasive ductal carcinoma, lobular carcinoma, mucinous carcinoma);
- Histological grade (Scarff–Bloom–Richardson classification, grades 1–3);
- TNM clinical staging: including T stage (tumor size and extension) and N stage (lymph node involvement), based on initial imaging and clinical exam prior to NAST;
- Hormonal receptor expression: estrogen receptor (ER) and progesterone receptor (PR), determined by immunohistochemistry;
- HER2 status: assessed via immunohistochemistry and in situ hybridization;
- Ki-67 proliferation index;
- Molecular subtype: categorized as HER2-positive, triple-negative (ER-, PR-, HER2-), or hormone receptor-positive/HER2-negative;
- Presence of germline mutations: BRCA1, BRCA2, TP53, or others, when genetic testing had been performed;
- Neoadjuvant treatment regimen: including details on chemotherapy (anthracyclines, taxanes), targeted therapy (trastuzumab, pertuzumab).

All clinical data were manually verified and curated to ensure consistency. These clinical features served as one of the three core input data types for the machine learning pipeline.

#### 2.2.2. Radiological Features

All pre-treatment breast MRI examinations were reviewed retrospectively by a board-certified radiologist with 4 years of experience, blinded to patient outcomes. The analysis was performed on a diagnostic workstation with access to multiplanar and multiparametric sequences. Standardized assessment criteria were applied to ensure consistency and reproducibility of annotations.

The radiologist performed a comprehensive qualitative and semi-quantitative evaluation of tumoral lesions according to the ACR BI-RADS^®^ MRI Atlas (5th Edition) guidelines [27], including the following parameters:

- Mass lesions: categorized based on shape (round, oval, irregular), margins (circumscribed, irregular, spiculated), and internal enhancement pattern (homogeneous, heterogeneous, rim enhancement);
- Non-mass enhancement (NME): characterized by distribution (focal, linear, segmental, regional, multiple regions, diffuse) and internal enhancement (homogeneous, heterogeneous, clumped, clustered ring);
- Parietal invasion: defined as direct contact or disruption of the anterior pectoral fascia, chest wall muscles;
- Tumor size: measured along the greatest dimension on T2-weighted axial images and early post-contrast T1-weighted axial images;
- Signal intensity on T2-weighted images: visually graded as hypointense, isointense, or hyperintense compared to surrounding normal fibroglandular breast tissue;
- Edema evaluation: the presence and type of associated edema on fat-saturated T2-weighted sequences were assessed and categorized as follows: absent, peritumoral edema (localized signal hyperintensity in subcutaneous fat or stroma adjacent to the lesion), pre-pectoral edema (signal hyperintensity in the space anterior to the pectoral muscle), and diffuse subcutaneous edema (extensive skin and fat stranding involving at least one quadrant).

All assessments were recorded in a structured spreadsheet and later converted into categorical or continuous variables for integration into the machine learning pipeline. In case of ambiguity or technical limitation in an image, a second radiologist was consulted for consensus reading.

#### 2.2.3. Radiomic Features

Radiomic features were extracted from conventional pre-treatment breast MRI sequences, specifically axial T2-weighted images and early-phase axial T1-weighted dynamic contrast-enhanced (DCE) images, which were selected to maximize comparability across MRI vendors and centers. All tumor segmentations were previously generated (see Section 2.3), and radiomic analysis was conducted using the Olea Sphere^®^ Texture Analysis package (Olea Medical, La Ciotat, France) and following the Image Biomarker Standardization Initiative (IBSI) guidelines.

To ensure reproducibility and minimize scanner-related variability, the following preprocessing steps were systematically applied to all input images before feature extraction:

- Histogram normalization: to rescale intensity values to a common dynamic range across patients and devices, reducing bias from contrast injection timing or scanner calibration.
- Voxel size resampling: all volumes were resampled to isotropic voxels of 1.0 mm × 1.0 mm × 1.0 mm using linear interpolation to ensure spatial consistency and allow accurate shape and texture analysis.
- Intensity discretization: grey-level values were quantized using a fixed bin number (64 bins), with relative discretization strategy (Lloyd–Max algorithm) applied separately for each sequence, allowing consistent texture calculation while accounting for intra-tumoral heterogeneity.

For each patient and each sequence (T1 and T2), a total of 108 radiomic features were extracted and categorized as follows:

- First-order histogram features (*n* = 19): describing global intensity distribution (e.g., mean, standard deviation, skewness, kurtosis, percentiles);
- Shape-based features (*n* = 17): quantifying geometric properties of the tumor volume (e.g., volume, surface area, sphericity, compactness, elongation);
- Texture features (*n* = 72): computed from five matrices—GLCM (Gray-Level Co-occurrence Matrix), GLRLM (Run-Length Matrix), GLSZM (Size Zone Matrix), NGTDM (Neighborhood Grey-Tone Difference Matrix), and GLDM (Dependence Matrix)—to characterize intra-tumoral heterogeneity and spatial relationships.

All extracted features were exported into structured CSV files and further processed in Python (v3.10) for harmonization and integration into the machine learning pipeline. Feature definitions and formulas followed IBSI conventions.

### 2.3. Tumor Segmentation Protocol

Tumor segmentation was performed manually and semi-automatically by a single board-certified radiologist with 4 years of experience, blinded to patient outcomes and pathological results. The segmentation process was carried out using the Olea Sphere^®^ BreastApp module (Olea Medical, La Ciotat, France). Prior to the segmentation work, the radiologist underwent dedicated training on the software tool with an Olea Medical engineer and completed a hands-on familiarization phase involving 20 patients in collaboration with a second radiologist, followed by another 10 patients reviewed jointly with a third radiologist. The radiologist worked closely with both colleagues throughout the entire workflow—from image loading to feature extraction—ensuring consistency and methodological rigor.

The primary segmentation was conducted on the early post-contrast T1-weighted dynamic sequence, using a semi-automatic thresholding algorithm available in Olea Sphere software (v3.0). The algorithm used a 70% enhancement threshold based on the peak signal intensity map derived from dynamic contrast-enhancement kinetics. This method allowed automated identification of the most vascularized regions of the tumor, typically corresponding to viable and enhancing tissue. The segmented volume of interest (VOI) was post-processed using a morphological closing operation, which enabled the inclusion of peripheral or non-enhancing intratumoral areas—such as necrosis or fibrosis—that may not reach the 70% enhancement threshold but still belong to the tumor core. The resulting VOI from the T1 post-contrast sequence was then replicated and co-registered onto the T2-weighted axial sequence, ensuring spatial consistency between functional (T1) and anatomical (T2) data. Manual corrections were applied when necessary to account for differences in tumor conspicuity or patient positioning, particularly in large tumors or cases with motion artifacts. All segmentations were visually validated slice-by-slice before being used for radiomic analysis.

Segmentations were stored in DICOM-RT format and exported directly from Olea Sphere to ensure compatibility with the radiomics extraction pipeline. Each segmented VOI was used as a mask to extract quantitative imaging features from both T1 and T2 sequences.

### 2.4. MRI Acquisition and Heterogeneity

As a regional oncological referral center, the study cohort included breast MRI examinations acquired on a variety of imaging platforms, reflecting real-world clinical heterogeneity. All pre-therapeutic MRIs were archived in our institutional Picture Archiving and Communication System (PACS) and reviewed for inclusion based on sequence completeness and image quality. Eligible examinations consistently included axial T2-weighted sequences and dynamic contrast-enhanced T1-weighted sequences, allowing standardization of feature extraction across the dataset.

In total, the imaging data encompassed examinations from 17 different MRI scanners, across four vendors (GE, Siemens, Philips, and Canon), and various magnetic field strengths (1.0 T, 1.5 T, and 3.0 T). This diversity of imaging platforms was leveraged intentionally to enhance the generalizability and external validity of our radiomics-based machine learning models. All MRI systems and their respective exam counts are summarized in Table 2.

### 2.5. Data Analysis

#### 2.5.1. Train-Test Split

To ensure a robust evaluation of model generalizability in the presence of imaging heterogeneity, we implemented a non-randomized stratified splitting strategy based on MRI scanner identity rather than purely random patient allocation. Patients were grouped according to the MRI machine model on which their pre-treatment imaging was acquired. Training and test datasets were then assembled to include distinct but balanced sets of scanner models, thereby simulating real-world deployment scenarios where radiomic models may encounter images from previously unseen scanners. Approximately 70% were used for training and 30% for testing, following commonly accepted practice.

This approach allowed us to assess the cross-scanner robustness of our predictive models and mitigate the risk of overfitting to scanner-specific signal characteristics. Care was taken to maintain a comparable distribution of clinical subtypes and molecular profiles across the training and testing cohorts. The final training and test sets included a representative mix of 1.5 T and 3.0 T systems and vendor types.

#### 2.5.2. Data Preprocessing

All clinical and radiological data were preprocessed to ensure compatibility with machine learning models. Categorical variables (e.g., molecular subtype, BI-RADS descriptors) were transformed into binary indicator variables using one-hot encoding, while ordinal variables (e.g., T stage, histological grade) were converted into numerical ranks.

Radiomic features were normalized using the ComBat harmonization algorithm [28,29,30], with the scanner model specified as the batch variable. This method effectively reduces inter-scanner variability while preserving biologically relevant information, thereby improving model generalizability across the 17 MRI systems included in this study.

All preprocessing steps were performed in Python using standard packages (Scikit-learn, Pandas), and were applied identically to both training and test sets.

#### 2.5.3. Pipeline Optimization

**Figure 2:**
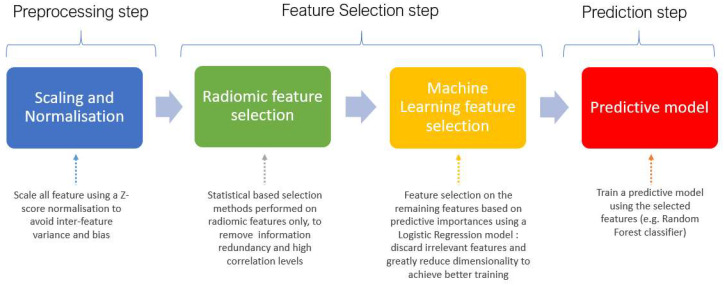
Overview of the machine learning pipeline applied. The process includes feature scaling and normalization, radiomic feature selection using statistical methods, machine learning-based feature selection using logistic regression importance scores, and final model training using the selected features (e.g., Random Forest classifier).

We implemented a supervised machine learning pipeline to automate predictive modeling and systematically evaluate combinations of input features, feature selection methods, and classifiers (Figure 2). The pipeline underwent two sequential optimization phases to maximize performance and reduce overfitting.

##### Pipeline Optimization 1 (PO1)

We performed broad hyperparameter tuning using 5-fold stratified cross-validation on the training set, with the ROC AUC as the primary optimization metric due to its robustness to class imbalance. While accuracy is a commonly reported metric, it may be misleading in the presence of class imbalance. Therefore, we selected the ROC AUC as the primary performance metric, as it provides a threshold-independent assessment of the classifier’s ability to distinguish between classes, regardless of their relative proportions. The following components were tested:

- Feature subsets: all combinations of Radiomics, Radiological, and Clinical features;
- Classification models: Logistic Regression, Support Vector Machines (SVM), Random Forest, Bagging Classifier, and K-Nearest Neighbors classifiers;
- Feature selection methods: Fisher score, ANOVA selection, MRMR (Maximum Relevance Minimum Redundancy selection), reliefF scoring test for supervised methods, and PCA decomposition for unsupervised selection.

##### Pipeline Optimization 2 (PO2)

To mitigate overfitting, PO2 applied a narrower search space based on the best-performing settings from PO1. We fixed feature selection and model settings, training Random Forest and SVM models using Fisher score-based selection, which demonstrated strong validation scores in the first iteration. The following hyperparameters were optimized:

■ Classifier hyperparameters:
  ○ For Random Forest models: maximum tree depth, minimum samples per split, and per leaf;
  ○ For SVM models: kernel type (linear or RBF) and regularization parameter.
■ Selection hyperparameters: feature retention count and feature subset selection (including Radiological features).

**Figure 3:**
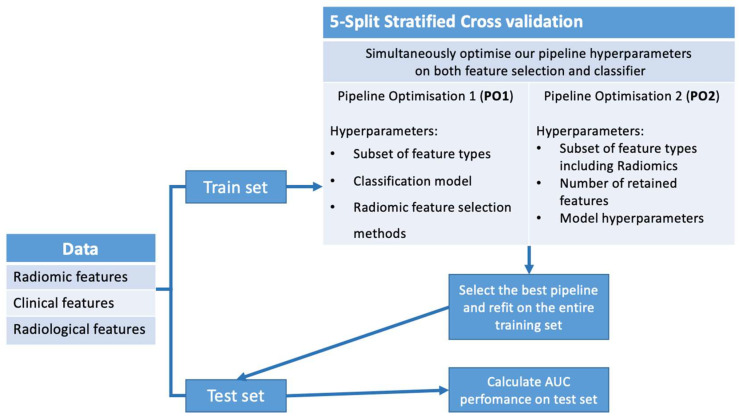
Two-stage hyperparameter optimization using 5-fold stratified cross-validation. PO1 explored feature types, classifiers, and selection methods. PO2 refined model and selection parameters. Final performance is assessed on the test set using AUC.

All optimization steps were performed using Python (Scikit-learn library) with nested cross-validation to avoid data leakage. For Random Forest, we optimized n_estimators (100–500), max_depth (5–50), min_samples_split (2–10), and min_samples_leaf (1–5). For SVM, we tested both linear and RBF kernels, with C ranging from 0.01 to 100 and gamma from 0.0001 to 1 (RBF only). Hyperparameters were tuned using nested cross-validation with the ROC AUC as the primary metric. A schematic summary of both optimization stages is provided in Figure 3.

## 3. Results

### 3.1. Results of PO1

PO1 was applied to various data subsets and their combinations to predict pCR. We evaluated model performance across various feature subsets using 5-fold cross-validation. Results are reported as mean AUC ± standard deviation in Table 3. The best performance was achieved using radiomic features alone (AUC 0.681 ± 0.049), followed closely by the combination of radiomic and clinical features (AUC 0.668 ± 0.046). Models relying solely on clinical or radiological features performed less accurately.

In addition to comparing different feature subsets (Table 3), we also assessed the impact of various feature selection techniques on model performance when using radiomic data alone. Table 4 presents the results obtained during Pipeline Optimization 1 (PO1), where we evaluated various feature selection methods applied exclusively to the radiomic feature subset, using a Support Vector Machine (SVM) classifier as the baseline model.

### 3.2. Prediction of pCR

**Figure 4:**
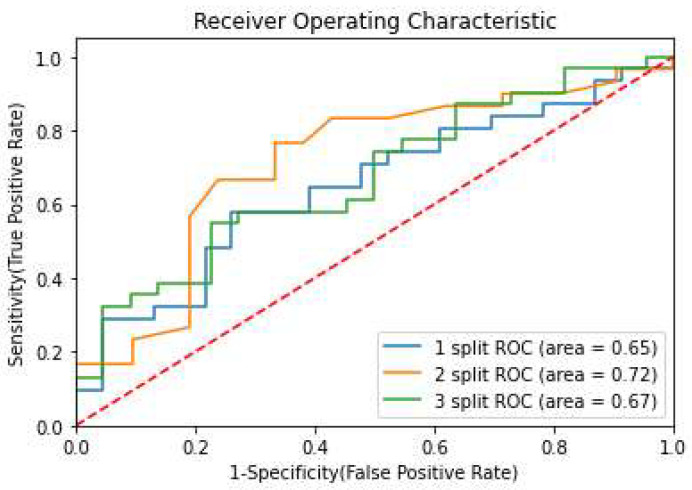
Prediction of pCR: ROC curves over 3 different test splits.

We applied PO2 with a Random Forest classifier across three different test splits, representing distinct imaging machine batches. The models achieved a maximum AUC score of 72% (95% CI: 59–85%) across splits, with a mean AUC exceeding 68% (95% CI: 55–81%) **(**Figure 4). Table 5 presents the top-performing features from each data type.

### 3.3. Prediction of pCR in Different Breast Cancer Subgroups

**Figure 5:**
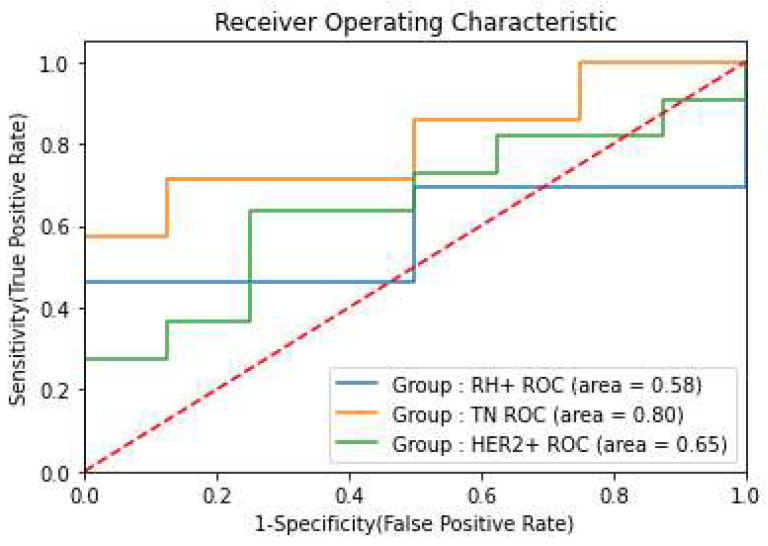
Prediction of pCR: ROC curves of best models in each subgroup.

Given the heterogeneous nature of breast cancer and its varying prognosis based on molecular profile, we stratified the cohort into three subgroups: HER2-positive (HER2+), hormone receptor-positive/HER2-negative (HR+), and triple-negative breast cancer (TN). We evaluated model performance across these subgroups to identify which patient populations demonstrated the most reliable prognostic predictions. PO2 with a SVM classifier showed superior performance in triple-negative and HER2-positive groups (Figure 5).

### 3.4. MRI Response Performance

A radiologist assessed radiological complete response (rCR), defined as absence of residual MRI enhancement on post-therapeutic images, when post-treatment MRI was available. Conventional wisdom suggests rCR as the optimal method for predicting pCR. Using true positive (TP), true negative (TN), false positive (FP), and false negative (FN) values to denote pCR prediction rates based on MRI response, the test set accuracy approximated (TP+TN)/ (TP+FP+TN+TP) = 81% (95% CI: 73–89%).

The equivalent AUC score for this statistical inference (assuming 100% confidence) reached 80.2% (95% CI: approx. 73–87%), substantially outperforming our initial models. Notably, our previous models relied solely on pre-treatment data, presenting an inherently greater challenge.

**Figure 6:**
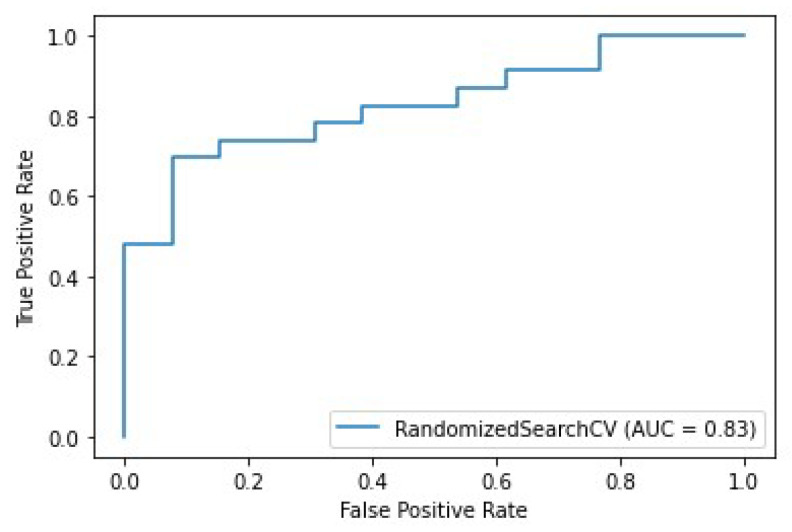
Prediction of pCR: ROC curves of the top model using the feature rCR.

We further evaluated this correlation by incorporating rCR into a Machine Learning pipeline. PO2 with a RandomForest classifier improved the AUC score on the same test set to 83% (95% CI: 76–89%) (Figure 6).

### 3.5. Prediction of Recurrence-Free Survival (RFS)

Among the 235 study participants, 37 patients (15.7%, 95% CI: 11.1–20.4%) experienced local recurrence during the follow-up period. We analyzed survival rates by predicting recurrence-free duration, a challenging task given the class imbalance and limited number of recurrence events in the population. We used bootstrapping to derive confidence intervals for AUC scores and Kaplan–Meier survival analysis with log-rank testing for recurrence-free survival.

**Figure 7:**
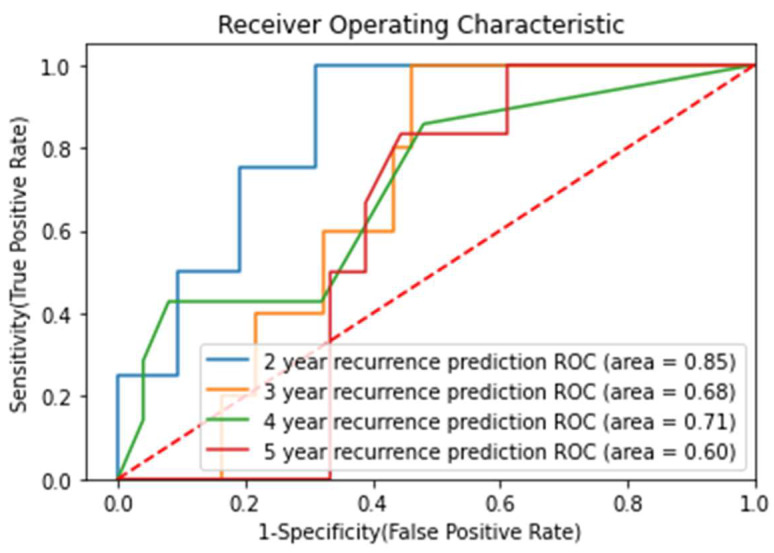
Prediction of RFS: ROC curves of the top model depending on the endpoint.

Given the varying follow-up times among patients, evaluating absolute recurrence risk or duration proved impractical. Instead, we approached this as a binary classification problem by establishing multiple endpoints at specific durations to assess patients’ recurrence status. We excluded patients with short follow-up times from higher endpoints. The analysis focused on 2-, 3-, 4-, and 5-year endpoints, which demonstrated progressively higher recurrence percentages in the population. We trained separate machine learning algorithms for each endpoint using PO2 and a Random Forest classifier (Figure 7).

Model performance decreased with longer endpoints, primarily due to severe class imbalance at earlier endpoints and the AUC score’s potential overoptimism in imbalanced classification scenarios (Table 6).

**Figure 8:**
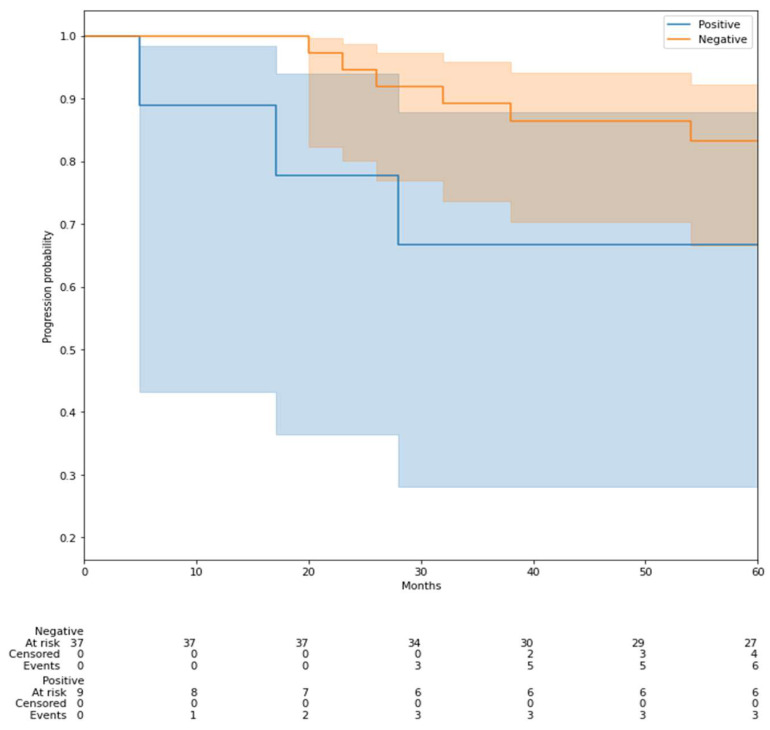
Kaplan–Meier survival curve predicted with our model using 2-year endpoint.

We fitted a Kaplan–Meier survival curve to the largest test set (corresponding to the 2-year endpoint) to evaluate the model’s effectiveness in identifying high-risk recurrence patients (Figure 8).

Although the log-rank test did not reach statistical significance, the Kaplan–Meier survival curve illustrates that patients classified as high-risk by our model had a trend toward earlier and more frequent recurrences. This suggests that the model may have clinical relevance for recurrence risk stratification, even in the presence of limited events and follow-up duration.

### 3.6. Additional Results

Although not a primary endpoint, we investigated prediction capabilities across different breast cancer subgroups (triple-negative, HR+/HER2-negative, and HER2-positive) using PO2 and an SVM classifier. The analysis yielded encouraging preliminary results for triple-negative and HR-positive subgroups (detailed results presented in Appendix A).

## 4. Discussion

This study evaluated machine learning models’ ability to predict pCR and RFS using pre-treatment MRI and clinical features in non-metastatic breast cancer patients receiving neoadjuvant systemic therapy. We carefully designed our models to align with daily diagnostic radiology practice, incorporating patients referred with previously performed MRI from different institutions and utilizing commercial software for semi-automatic segmentation and feature extraction.

Our radiomics-based models demonstrated superior prediction ability, achieving a maximum AUC of 0.72, with optimal performance observed in triple-negative (AUC 0.80) and HER2-positive (AUC 0.65) subgroups. All models underwent training and testing using data from various MRI machines, confirming the results’ independence from specific MRI equipment. When comparing our models with radiologic complete response (rCR), we found rCR maintained superior performance (AUC 0.81), and incorporating this feature into our models provided minimal additional benefit (AUC 0.83).

RFS prediction proved more challenging due to limited follow-up duration, small sample size, and class imbalance. Although the Kaplan–Meier curves indicated insufficient statistical power, the observed AUC values showed promise for future clinical studies. In particular, the Kaplan–Meier analysis of the 2-year endpoint (Figure 8) demonstrated a visible separation between predicted high- and low-risk groups, supporting the potential of the model to stratify recurrence risk despite the lack of statistical significance.

To contextualize our results, we compiled a comparative summary of recent studies evaluating machine learning models for pCR prediction using radiomics-based approaches (Table 7). Our findings align with previous research on pre-treatment data [21,22,23,24,25]. Liu et al. [24] reported an AUC of 0.79 for pCR prediction, with superior performance in triple-negative and HR-positive subgroups, while our results showed better performance in triple-negative and HER2-positive subgroups. Their multicenter study employed external validation sets but did not assess clinical outcomes. Braman et al. [22] achieved an AUC of 0.74 for pCR prediction using intratumoral and peritumoral radiomics features, though their study had a smaller cohort (117 patients) and excluded clinical or radiological features from their analysis, similar to other studies [25]. Bitencourt et al. [31] reported an MRI-based model for pCR prediction in HER2-positive tumors with 83.9% accuracy, supporting our findings.

Notably, few studies have evaluated clinical outcome prediction such as RFS or compared their radiomic signatures with radiologic response. While these models might predict outcomes before NAST initiation, radiological response remains crucial for pCR prediction, making its integration into clinical prediction models essential. Early mid-treatment MRI [32,33,34,35] combined with radiomics biomarkers could offer a viable strategy for optimizing treatment sequencing in breast cancer patients.

Our study faced several limitations such as sample size, its retrospective single-center design and heterogeneous imaging protocols. First, the heterogeneous data encompassing over 17 different MRI models may have enhanced result generalizability but potentially impacted overall model performance. Second, diffusion sequences, known for their valuable contribution to radiomics [24,36], were limited in availability. Additionally, several inherent radiomics-related challenges warrant mention. The semi-automatic segmentation process, while involving radiologist oversight, lacked rigorous reproducibility testing [37]. Model hyperparameters, particularly feature subset selection, varied across training splits, indicating that individual radiomic features alone lack prognostic power. Moreover, the feature importance rankings reveal that many radiomic features lack straightforward interpretability [38], potentially complicating their integration into radiologists’ diagnostic processes. No external validation cohort meaning that the models were evaluated on an internal test set. Future work should include multi-center external validation to confirm generalizability. Nevertheless, these findings could inform future clinical trials investigating pCR prediction in breast cancer patients using innovative radiomics and clinical feature approaches. While our machine learning pipeline shares similarities with previously published frameworks in other clinical contexts [39,40], it was specifically tailored to the breast cancer setting. The choice of features, outcome definitions, and harmonization strategies was driven by the biological behavior of the disease and real-world diagnostic constraints. This underlines the importance of adapting such models to the clinical context to ensure both robustness and relevance.

## 5. Conclusions

Our study demonstrates that machine learning models show encouraging results for pCR prediction using clinical and pre-treatment MRI data, particularly in triple-negative and HER2-positive breast cancer subgroups. Despite our models’ promising performance, radiological complete response currently remains the optimal pCR predictor following NAST, pending further validation of ML models in additional studies. Future research should focus on investigating these techniques’ ability to predict survival outcomes, particularly RFS, in larger studies with extended follow-up periods.

## Figures and Tables

**Table 1:** Demographic characteristics of the cohort.

Characteristics	Value
Age at diagnosis (years, mean ± SD)	46.47 ± 11.56
Histologic subtype	
■ Invasive ductal carcinoma	208 (88.5%)
■ Lobular carcinoma	14 (6%)
■ Mucinous adenocarcinoma	2 (0.9%)
■ Metaplastic carcinoma	2 (0.9%)
■ Neuroendocrine tumor	2 (0.9%)
■ Others	5 (2.8%)
Histological grade	
■ 1	9 (3.8%)
■ 2	90 (38.2%)
■ 3	130 (55.3%)
■ Undetermined	6 (2.7%)
Molecular subtype	
■ Her2+	91 (38.8%)
■ Her2−/Hormone receptor +	72 (30.6%)
■ Her2−/Hormone receptor −	72 (30.6%)
Genetic mutation	
■ None	214 (91%)
■ BRCA1	9 (3.7%)
■ BRCA2	8 (3.5%)
■ TP53	4 (1.8%)
T staging	
■ 1	4 (1.8%)
■ 2	138 (58.7%)
■ 3	71 (30.2%)
■ 4a	5 (2.1%)
■ 4b	4 (1.8%)
■ 4d	13 (5.4%)
Lymph node invasion	135 (57%)

**Table 2:** MRI models used in the cohort.

MRI Models	Constructor	MRI Field (Tesla)	Exams Number
OptimaMR450w	GE	1.5	61 ^a^
Aera	Siemens	1.5	53
DiscoveryMR750w	GE	3	36 ^b^
Avanto	Siemens	1.5	18
OptimaMR360	GE	1.5	13
Spectra	Siemens	3	11
SignaHDxt	GE	1.5	11
Ingenia	Philips	3	7
PanoramaHFO	Philips	1	5
Essenza	Siemens	1.5	4
Amira	Siemens	1.5	3
Achieva	Siemens	1.5	3
Titan	Canon	1.5	2
Skyra	Siemens	3	2
Signa Explorer	GE	1.5	2
Signa Excite	GE	1.5	1
Signa Architect	GE	3	1
Undetermined		1.5	2

^a^ A total of 46 from Gustave Roussy radiology department. ^b^ A total of 30 from Gustave Roussy radiology department.

**Table 3:** Performance (AUC) of the best models depending on the feature subset, reported as mean ± standard deviation over 5-fold cross-validation during Pipeline Optimization 1 (PO1).

Subset of Feature	AUC (mean +/− SD)
Clinical	0.603 ± 0.042
Radiologic	0.583 ± 0.038
Radiologic + Clinical	0.646 ± 0.044
Radiomic	0.681 ± 0.049
Radiomic + Clinical	0.668 ± 0.046
Radiomic + Radiologic	0.664 ± 0.051
Radiomic + Clinical + Radiologic	0.650 ± 0.045

**Table 4:** Performance of feature selection methods (radiomics only, PO1, SVM classifier).

Technique of Feature Selection	Mean F1 Score	Mean AUC Score
Fischer	0.771	0.669
MRMR	0.782	0.662
ANOVA	0.777	0.655
TSCR	0.771	0.643
ReliefF	0.770	0.640
PCA	0.764	0.601

**Table 5:** Top-ranked features contributing to pCR prediction in the best-performing model (Pipeline Optimization 2, Random Forest classifier, combined radiomic, radiologic, and clinical data).

Radiomic Features	Importance
T2-weighted: original first-order 90th percentile	0.101
T2- weighted: original first-order variance	0.081
T2-weighted: original first-order 10th percentile	0.080
T2-weighted: Original Grey Level Size Zone Matrix Grey Level Variance	0.074
T2-weighted: original first-order minimum	0.065
**Radiologic features**	**Importance**
Axial diameter on T1-weighted enhanced	0.088
Peri-tumoral oedema	0.026
Irregular margin	0.013
**Clinical features**	**Importance**
Age at diagnosis	0.049
No hormonal receptor expression Histologic grade	0.036 0.011

**Table 6:** Number and percentage of the relevant samples in the test set.

Endpoint (Years)	Number of Recurrence	Number of Samples	Recurrence Percentage
2	4	46	8.7
3	5	42	11.9
4	7	32	21.8
5	6	24	25

**Table 7:** Comparative summary of similar studies on pCR prediction using machine learning and radiomics.

Study (First Author, Year)	Cohort Size	Input Data	ML Method	Reported AUC	External Validation	Main Limitations
Liu et al., 2019 [24]	364	Multiparametric MRI (radiomics)	SVM	0.79	Yes	No integration of clinical outcomes
Braman et al., 2017 [22]	117	Intratumoral and peritumoral MRI features	Logistic Regression	0.74	No	Small cohort, radiomics only
Bitencourt et al., 2020 [32]	93	MRI-based features	Machine learning	0.84 (Accuracy)	No	No radiomics or clinical variable comparison
Cain et al., 2019 [25]	288	MRI radiomics	SVM, Random Forest	0.73	Yes	Limited integration of clinical features
Current Study (Hajri et al.)	235	Clinical, radiologic, radiomic MRI	Random Forest, SVM	0.72	No	No external validation, limited DWI availability

**Figure A1:**
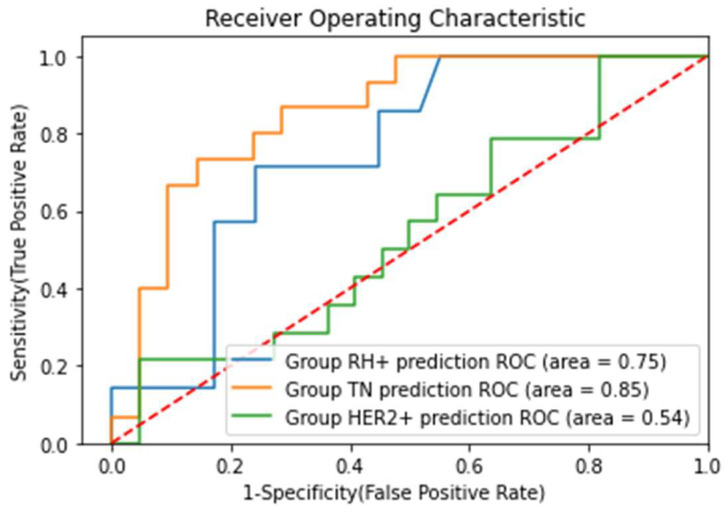
ROC curves of the best models per subgroup.

## Data Availability

The original contributions presented in this study are included in the article; further inquiries can be directed to the corresponding author.

## Abbreviations

AUC	Area Under the Receiver Operating Characteristic Curve
BI-RADS	Breast Imaging Reporting and Data System
DCE	Dynamic Contrast-Enhanced
ER	Estrogen receptor
GLDM	Gray Level Dependence Matrix
GLCM	Gray Level Co-occurrence Matrix
GLRLM	Gray Level Run Length Matrix
GLSZM	Gray Level Size Zone Matrix
HER2	Human Epidermal Growth Factor Receptor 2
IBSI	Image Biomarker Standardization Initiative
IHC	Immunohistochemistry
KNN	K-Nearest Neighbors
MRIMagnetic	Resonance Imaging
MRMR	Maximum Relevance Minimum Redundancy
NME	Non-mass enhancement
PCA	Principal Component Analysis
PR	Progesterone Receptor
pCR	Pathological complete response
PO1	Pipeline Optimization 1
PO2	Pipeline Optimization 2
RF	Random Forest
ROC	Receiver Operating Characteristic
RBF	Radial Basis Function
ROI	Region of Interest
SBR	Scarff–Bloom–Richardson (grading system)
SVM	Support Vector Machine
T1w	T1-weighted
T2w	T2-weighted
TNM	Tumor Node Metastasis (staging system)
VOI	Volume of interest

## Appendix A. Prediction of Molecular Subtypes of Breast Cancer

We approached this problem as a binary classification task, aiming to determine which groups are most distinguishable from others. This required training three separate models in a one-vs-all fashion, with each model predicting a specific group (designated as the positive class).

Figure A1 summarizes our results, demonstrating better performance in identifying triple-negative and hormone receptor-positive groups. While technically and scientifically interesting, this finding has limited clinical relevance, as biopsy will remain the gold standard for molecular subtyping for the foreseeable future.

## References

[1] Sung H., Ferlay J., Siegel R.L., Laversanne M., Soerjomataram I., Jemal A., Bray F. (2021). Global cancer statistics 2020: GLOBOCAN estimates of incidence and mortality worldwide for 36 cancers in 185 countries. CA Cancer J. Clin..

[2] Gianni L., Pienkowski T., Im Y.-H., Roman L., Tseng L.-M., Liu M.-C., Lluch A., Staroslawska E., de la Haba-Rodriguez J., Im S.-A. (2012). Efficacy and safety of neoadjuvant pertuzumab and trastuzumab in women with locally advanced, inflammatory, or early HER2-positive breast cancer (NeoSphere): A randomised multicentre, open-label, phase 2 trial. Lancet Oncol..

[3] Mieog J.S.D., van der Hage J.A., van de Velde C.J.H. (2007). Neoadjuvant chemotherapy for operable breast cancer. Br. J. Surg..

[4] Symmans W.F., Peintinger F., Hatzis C., Rajan R., Kuerer H., Valero V., Assad L., Poniecka A., Hennessy B., Green M. (2007). Measurement of residual breast cancer burden to predict survival after neoadjuvant chemotherapy. J. Clin. Oncol..

[5] Cortazar P., Zhang L., Untch M., Mehta K., Costantino J.P., Wolmark N., Bonnefoi H., Cameron D., Gianni L., Valagussa P. (2014). Pathological complete response and long-term clinical benefit in breast cancer: The CTNeoBC pooled analysis. Lancet.

[6] Masuda N., Lee S.-J., Ohtani S., Im Y.-H., Lee E.-S., Yokota I., Kuroi K., Im S.-A., Park B.-W., Kim S.-B. (2017). Adjuvant capecitabine for breast cancer after preoperative chemotherapy. N. Engl. J. Med..

[7] von Minckwitz G., Huang C.-S., Mano M.S., Loibl S., Mamounas E.P., Untch M., Wolmark N., Rastogi P., Schneeweiss A., Redondo A. (2019). Trastuzumab emtansine for residual invasive HER2-positive breast cancer. N. Engl. J. Med..

[8] Montemurro F., Nuzzolese I., Ponzone R. (2020). Neoadjuvant or Adjuvant Chemotherapy in Early Breast Cancer?. Expert Opin. Pharmacother..

[9] Heil J., Kuerer H.M., Pfob A., Rauch G., Sinn H.P., Golatta M., Liefers G.J., Vrancken Peeters M.J. (2020). Eliminating the breast cancer surgery paradigm after neoadjuvant systemic therapy: Current evidence and future challenges. Ann. Oncol..

[10] Garland M.L., Vather R., Bunkley N., Pearse M., Bissett I.P. (2014). Clinical tumour size and nodal status predict pathologic complete response following neoadjuvant chemoradiotherapy for rectal cancer. Int. J. Color. Dis..

[11] Hylton N.M., Blume J.D., Bernreuter W.K., Pisano E.D., Rosen M.A., Morris E.A., Weatherall P.T., Lehman C.D., Newstead G.M., Polin S. (2012). Locally advanced breast cancer: MR imaging for prediction of response to neoadjuvant chemotherapy—Results from ACRIN 6657/I-SPY TRIAL. Radiology.

[12] Shin H.J., Kim H.H., Ahn J.H., Kim S.-B., Jung K.H., Gong G., Son B.H., Ahn S.H. (2011). Comparison of mammography, sonography, MRI and clinical examination in patients with locally advanced or inflammatory breast cancer who underwent neoadjuvant chemotherapy. Br. J. Radiol..

[13] Cao Y., Wang X., Li L., Shi J., Zeng X., Huang Y., Chen H., Jiang F., Yin T., Nickel D. (2023). Early prediction of pathologic complete response of breast cancer after neoadjuvant chemotherapy using longitudinal ultrafast dynamic contrast-enhanced MRI. Diagn. Interv. Imaging.

[14] Rella R., Bufi E., Belli P., Petta F., Serra T., Masiello V., Scrofani A.R., Barone R., Orlandi A., Valentini V. (2020). Association between background parenchymal enhancement and tumor response in patients with breast cancer receiving neoadjuvant chemotherapy. Diagn. Interv. Imaging.

[15] Avanzo M., Stancanello J., El Naqa I. (2017). Beyond imaging: The promise of radiomics. Phys. Med..

[16] Song J., Yin Y., Wang H., Chang Z., Liu Z., Cui L. (2020). A review of original articles published in the emerging field of radiomics. Eur. J. Radiol..

[17] Aerts H.J.W.L. (2016). The potential of radiomic-based phenotyping in precision medicine: A review. JAMA Oncol..

[18] Papanikolaou N., Matos C., Koh D.M. (2020). How to develop a meaningful radiomic signature for clinical use in oncologic patients. Cancer Imaging.

[19] Lambin P., Leijenaar R.T.H., Deist T.M., Peerlings J., de Jong E.E.C., van Timmeren J., Sanduleanu S., Larue R.T.H.M., Even A.J.G., Jochems A. (2017). Radiomics: The bridge between medical imaging and personalized medicine. Nat. Rev. Clin. Oncol..

[20] Parmar C., Grossmann P., Bussink J., Lambin P., Aerts H.J.W.L. (2015). Machine Learning Methods for Quantitative Radiomic Biomarkers. Sci. Rep..

[21] Granzier R.W.Y., van Nijnatten T.J.A., Woodruff H.C., Smidt M.L., Lobbes M.B.I. (2019). Exploring Breast Cancer Response Prediction to Neoadjuvant Systemic Therapy Using MRI-Based Radiomics: A Systematic Review. Eur. J. Radiol..

[22] Braman N.M., Etesami M., Prasanna P., Dubchuk C., Gilmore H., Tiwari P., Plecha D., Madabhushi A. (2017). Intratumoral and Peritumoral Radiomics for the Pretreatment Prediction of Pathological Complete Response to Neoadjuvant Chemotherapy Based on Breast DCE-MRI. Breast Cancer Res..

[23] Tahmassebi A., Wengert G.J., Helbich T.H., Bago-Horvath Z., Alaei S., Bartsch R., Dubsky P., Baltzer P., Clauser P., Kapetas P. (2019). Impact of Machine Learning With Multiparametric Magnetic Resonance Imaging of the Breast for Early Prediction of Response to Neoadjuvant Chemotherapy and Survival Outcomes in Breast Cancer Patients. Invest. Radiol..

[24] Liu Z., Li Z., Qu J., Zhang R., Zhou X., Li L., Sun K., Tang Z., Jiang H., Li H. (2019). Radiomics of Multiparametric MRI for Pretreatment Prediction of Pathologic Complete Response to Neoadjuvant Chemotherapy in Breast Cancer: A Multicenter Study. Clin. Cancer Res..

[25] Cain E.H., Saha A., Harowicz M.R., Marks J.R., Marcom P.K., Mazurowski M.A. (2019). Multivariate Machine Learning Models for Prediction of Pathologic Response to Neoadjuvant Therapy in Breast Cancer Using MRI Features: A Study Using an Independent Validation Set. Breast Cancer Res. Treat..

[26] Doran S.J., Kumar S., Orton M., d’Arcy J., Kwaks F., O’Flynn E., Ahmed Z., Downey K., Dowsett M., Turner N. (2021). “Real-World” Radiomics from Multi-Vendor MRI: An Original Retrospective Study on the Prediction of Nodal Status and Disease Survival in Breast Cancer, as an Exemplar to Promote Discussion of the Wider Issues. Cancer Imaging.

[27] Morris E.A., Comstock C.E., Lee C.H. (2013). ACR BI-RADS^®^ Magnetic Resonance Imaging. ACR BI-RADS^®^ Atlas, Breast Imaging Reporting and Data System.

[28] Da-ano R., Masson I., Lucia F., Doré M., Robin P., Alfieri J., Rousseau C., Mervoyer A., Reinhold C., Castelli J. (2020). Performance Comparison of Modified ComBat for Harmonization of Radiomic Features for Multicenter Studies. Sci. Rep..

[29] Orlhac F., Lecler A., Savatovski J., Goya-Outi J., Nioche C., Charbonneau F., Ayache N., Frouin F., Duron L., Buvat I. (2021). How Can We Combat Multicenter Variability in MR Radiomics? Validation of a Correction Procedure. Eur. Radiol..

[30] Saint Martin M.-J., Orlhac F., Akl P., Khalid F., Nioche C., Buvat I., Malhaire C., Frouin F. (2021). A Radiomics Pipeline Dedicated to Breast MRI: Validation on a Multi-Scanner Phantom Study. MAGMA.

[31] Bitencourt A.G., Gibbs P., Saccarelli C.R., Daimiel I., Gullo R.L., Fox M.J., Thakur S., Pinker K., Morris E.A., Morrow M. (2020). MRI-Based Machine Learning Radiomics Can Predict HER2 Expression Level and Pathologic Response after Neoadjuvant Therapy in HER2 Overexpressing Breast Cancer. EBioMedicine.

[32] Chen J.H., Feig B., Agrawal G., Yu H., Carpenter P.M., Mehta R.S., Nalcioglu O., Su M.Y. (2008). MRI Evaluation of Pathologically Complete Response and Residual Tumors in Breast Cancer after Neoadjuvant Chemotherapy. Cancer.

[33] Eun N.L., Kang D., Son E.J., Park J.S., Youk J.H., Kim J.-A., Gweon H.M. (2020). Texture Analysis with 3.0-T MRI for Association of Response to Neoadjuvant Chemotherapy in Breast Cancer. Radiology.

[34] Dave R.V., Millican-Slater R., Dodwell D., Horgan K., Sharma N. (2017). Neoadjuvant Chemotherapy with MRI Monitoring for Breast Cancer. Br. J. Surg..

[35] Parikh J., Selmi M., Charles-Edwards G., Glendenning J., Ganeshan B., Verma H., Mansi J., Harries M., Tutt A., Goh V. (2014). Changes in Primary Breast Cancer Heterogeneity May Augment Midtreatment MR Imaging Assessment of Response to Neoadjuvant Chemotherapy. Radiology.

[36] Dong Y., Feng Q., Yang W., Lu Z., Deng C., Zhang L., Lian Z., Liu J., Luo X., Pei S. (2018). Preoperative Prediction of Sentinel Lymph Node Metastasis in Breast Cancer Based on Radiomics of T2-Weighted Fat-Suppression and Diffusion-Weighted MRI. Eur. Radiol..

[37] Zhao B., Tan Y., Tsai W.-Y., Qi J., Xie C., Lu L., Schwartz L.H. (2016). Reproducibility of Radiomics for Deciphering Tumor Phenotype with Imaging. Sci. Rep..

[38] Papadimitroulas P., Brocki L., Christopher Chung N., Marchadour W., Vermet F., Gaubert L., Eleftheriadis V., Plachouris D., Visvikis D., Kagadis G.C. (2021). Artificial Intelligence: Deep Learning in Oncological Radiomics and Challenges of Interpretability and Data Harmonization. Phys. Medica.

[39] Gibała S., Obuchowicz R., Lasek J., Piórkowski A., Nurzynska K. (2023). Textural Analysis Supports Prostate MR Diagnosis in PIRADS Protocol. Appl. Sci..

[40] Obuchowicz R., Nurzynska K., Pierzchala M., Piorkowski A., Strzelecki M. (2023). Texture Analysis for the Bone Age Assessment from MRI Images of Adolescent Wrists in Boys. J. Clin. Med..

